# Sustainability Analysis of Processes to Recycle Discharged Lithium-Ion Batteries, Based on the ESCAPE Approach

**DOI:** 10.3390/ma15238527

**Published:** 2022-11-30

**Authors:** Ario Fahimi, Alessandra Zanoletti, Antonella Cornelio, Elsayed Mousa, Guozhu Ye, Patrizia Frontera, Laura Eleonora Depero, Elza Bontempi

**Affiliations:** 1 INSTM and Chemistry for Technologies Laboratory, Department of Mechanical and Industrial Engineering, University of Brescia, Via Branze, 38, 25123 Brescia, Italy; 2 SWERIM AB, Aronstorpsvägen 1, SE-97437 Luleå, Sweden; 3 Central Metallurgical Research and Development Institute, Cairo 12422, Egypt; 4 INSTM and Department of Civil, Energy, Environmental and Material Engineering (DICEAM), University Mediterranea of Reggio Calabria, Via Graziella Loc. Feo di Vito, 89124 Reggio Calabria, Italy

**Keywords:** ESCAPE, lithium-ion batteries, embodied energy, carbon footprint

## Abstract

There are several recycling methods to treat discharged lithium-ion batteries, mostly based on pyrometallurgical and hydrometallurgical approaches. Some of them are promising, showing high recovery efficiency (over 90%) of strategic metals such as lithium, cobalt, and nickel. However, technological efficiency must also consider the processes sustainability in terms of environmental impact. In this study, some recycling processes of spent lithium-ion batteries were considered, and their sustainability was evaluated based on the ESCAPE “Evaluation of Sustainability of material substitution using CArbon footPrint by a simplifiEd approach” approach, which is a screening tool preliminary to the Life Cycle Assessment (LCA). The work specifically focuses on cobalt recovery comparing the sustainability of using inorganic or organic acid for the leaching of waste derived from lithium-ion batteries. Based on the possibility to compare different processes, for the first time, some considerations about technologies optimization have been done, allowing proposing strategies able to save chemicals. In addition, the energy mix of each country, to generate electricity has been considered, showing its influence on the sustainability evaluation. This allows distinguishing the countries using more low-carbon sources (nuclear and renewables) for a share of the electricity mix, where the recycling processes result more sustainable. Finally, this outcome is reflected by another indicator, the eco-cost from the virtual pollution model 99′ proposed by Vogtländer, which integrates the monetary estimation of carbon footprint.

## 1. Introduction

To mitigate climate change and obtain a fossil fuel-free economy, the global community has agreed that greenhouse gas (GHG) emissions must be significantly reduced [1]. This must be traduced in urgent and more intensive actions against climate change around the world. Electrification is one of the most affordable energy transitions and cost-effective ways to mitigate climate change by preventing or reducing GHG emissions. In this context, lithium-ion batteries (LIBs) appeared as one of the effective solutions in modern society to reduce the carbon dioxide generated by one of the major emitters, which is the mobility sector. LIBs are a viable opportunity to pursue the Paris Agreement [2], representing a critical pillar to achieving a fossil fuel-free economy. LIBs manufacturing is globally growing very fast to meet the high demand and usage in several vital applications such as electric vehicles (EVs), portable electronics, and grid storage. The International Energy Agency (IEA) reveals that the number of EVs will reach 245 million by 2030 [3]. It is estimated that the global market of LIBs will reach 139 billion dollars in 2026. 

This risky price surge is attributed to the fact that the global capacity of lithium (Li) mines and the ability to process lithium into batteries is expected to fall short [4]. 

Battery-cell manufacturing is overwhelmingly dominated by China, where more than 80% of all battery cells are manufactured. This is also due to the existence of more limited environmental and regulatory restrictions, in comparison to, for example, Europe.

The rapid growth in demand for LIBs will inevitably produce many spent batteries [5]. Furthermore, a battery’s lifetime is also limited (a few years). This implies an increase in the landfill world [6].

Consequently, great attention is being paid to secure the sustainability of the critical raw materials which are used in LIBs including cobalt (Co), Li, natural graphite, and phosphorus (P), in addition to other valuable metals such as nickel (Ni), copper (Cu), aluminium (Al), iron (Fe) and manganese (Mn). The high demand for these critical metals coupled with their limited availability represents a real risk for the sustainable manufacturing of LIBs and brings new challenges to modern society.

The most critical metal used for LIBs manufacturing is Co. It is generally extracted by corresponding minerals in the primary production of copper and nickel [7]. Most of the world’s Co supply (about 60%) is guaranteed by mining from the Democratic Republic of Congo, which holds approximately 34% of the world’s Co resources. The weight percentage of the metal in the minerals, is on average, around 2% [8], but following refining, it can reach 4–6% [9]. Clearly, the percentage of Co present in the mineral greatly affects the economics of the process, depending on the degree of purity of Co of the initially treated material.

Since the 1970s, the Co-market has been riddled with supply shortages relative to the demand, with great concerns arising because of the increasing demand for this metal in rechargeable LIBs [7,10]. From an economic point of view, Co has the highest commercial price among the metals used for cathodic mixed oxides production. For these reasons, a shift towards lower cobalt chemistries to produce battery cathodes was introduced, leading to the growth of nickel adoption instead. However, this is insufficient to reduce the Co supply vulnerability.

Discarded LIBs are classified as hazardous material, and suitable disposal of spent automotive batteries incurs substantial costs and poses different challenges to the LIBs industry. Recycling is potentially the most promising end-of-life management option because it has the capability to considerably reduce the environmental impacts of batteries, while simultaneously helping to mitigate price surges and supply disruptions of battery materials, especially when the recovered metals are inserted back into the battery supply chain (i.e., closed-loop recycled) [6,11].

Recycling can relieve the pressure on the primary supply. For example, the content of valuable metals, like Co and Li, in spent LIBs, is higher than in natural ores [12]. For bulk metals, recycling practices are well established, but this is not yet the case for many metals, involved in the energy transition, such as Co. Recycling would not eliminate the need for continued investment in new supplies. It is estimated that by 2040, recycled quantities of Cu, Li, Ni and Co from spent batteries could reduce primary supply requirements for these minerals by around 10% [13]. Some security benefits of recycling may be also achieved, resulting greater for wider deployment areas of clean energy technologies due to greater economies of scale.

The LIBs recycling processes generally start with the discharging of the batteries to reduce their hazard level resulting from residual stored energy, which could otherwise activate further reactions and overheating. The following steps are generally based on mechanical, pyrometallurgical, and hydrometallurgical treatments. These units aim to recover economically interesting materials from the electrodes of the LiBs cells. The negative electrode (anode) and the positive electrode (cathode) are the main components of a lithium-ion battery in terms of economic value. The anode is composed of a Cu foil coated with graphite. The cathode is an aluminium foil covered with an electrochemically active material, generally composed of transition metal oxides. The adhesion between the aluminium foil and the active material is improved by a polymeric binder, mostly polyvinylidene fluoride (PVDF). The electrodes are separated by a separator made from polypropylene and inserted in a case filled with electrolytes. The electrolyte is typically composed of a mixture of alkyl carbonates and lithium hexafluorophosphate (LiPF_6_). LiPF_6_ is used for the high conductivity of its organic solutions. 

As part of the European Green Deal, in 2020 a new legislative framework was proposed by the European Commission to replace the 2006 Battery Directive. The targets for recycling efficiencies were settled to 65% for LIBs and 75% for Pb-acid batteries by 2025. Moreover, the target material recovery rates of 95% for Co, Cu, Pb, Ni, and 70% for Li by 2030 have been proposed [14].

However, this could come across with process economics, which depends mostly on the recovery of Co, by far the most valuable element in the battery, and research is underway to reduce the quantity of Co used [15].

Highlighting the critical raw materials recovery potential can enhance productivity and increase the recyclability of these strategic materials. These activities require the establishment of suitable procedures able to account for the sustainability of a new process, aiming to maximize the recycling potential and support strategic choices in terms of selected technologies. However, some already existing instruments, devoted to evaluating the environmental impacts of technologies, such as life cycle assessment (LCA), have some disadvantages related to their complexity, multiple indicators use, and the need to set preliminary decisions, also with insufficient inventory data. They are often not applicable to emerging technologies, developed at low technology readiness levels [16].

In this frame, simplified tools to evaluate the sustainability of raw materials substitution have been recently developed, with the aim to propose suitable resource-saving alternatives. ESCAPE approach “Evaluation of Sustainability of material substitution using CArbon footPrint by a simplifiEd approach” [17], is used, as a sustainability evaluation tool, preliminary to LCA, before spending a long time in an onerous full-LCA study.

In the present paper, the literature, for a total of 10 recycling processes, discussing hydrometallurgical recycling methods using organic acids (lactic acid, citric acid, acetic acid) and inorganic acids (sulphuric acid, nitric acid, phosphoric acid) for leaching the BM of spent LIBs are considered. For the first time, an evaluation of the sustainability of the proposed processes has been realized, by using the ESCAPE approach. The novelty of this work not only consists in the possibility to evaluate and compare some technologies but also in the derived considerations about their optimization, allowing proposing strategies able to save chemicals (the most crucial contribution in a hydrometallurgical process) and increase the processes sustainability.

Before data analysis, a validation step for the case of Co as a reference process representing the state-of-the-art extraction is proposed. The ESCAPE approach also allows for deriving some considerations about the possibility to apply the proposed processes in different countries, since it accounts for the energy mix for each state.

Finally, eco-costs, which are a measure to express the amount of environmental burden of a product are also considered. They represent the (marginal) costs which should be taken to reduce the environmental pollution and materials depletion in our world to a level which is a bearable capacity of our Earth.

For this aim, the virtual pollution prevention cost 99′ method, proposed by Vogtländer [18], is considered.

## 2. Methods

### 2.1. Recycling of Spent LIBs

The aim of the recycling processes is to recover active material of valuable metals, such as Ni, Mn, Co, and Li from spent LIBS, which can be used to synthesize new precursors for cathodes of new batteries.

These elements could be recovered only after the pack cells have undergone physical treatments (shredding, grinding, heat treatment, density separation), to obtain a fine powder, generally called Black Mass (BM). BM is rich in strategic metals, but also in “impurities” which are deleterious for downstream recycling processes.

In most cases, LIBs undergo thermal treatment, e.g., pyrolysis, before mechanical treatment to reduce the energy content in a controlled way, to eliminate the organic components. This process usually takes place at around 500 °C and is limited by the melting point of Al (660 °C). Afterwards, the pyrolyzed batteries can be treated with a combination of mechanical treatment with pyrometallurgical and/or hydrometallurgical treatment [19], possibly after the electrolyte recovery [20].

This study was realized in the frame of ERA-MIN2 project, namely NEXT-LIB, which proposes an integrated and innovative approach to maximize valuable material recovery from spent LIBs, based on process integration combining innovative mechanical with efficient pyrometallurgical and hydrometallurgical processes. 

The ongoing research of the NEXT-LIB aims for upscaling of the pyro- and hydro-metallurgy treatment after graphite separation followed by evaluation of the product quality and executing the techno-economic and environmental analysis.

The NEXT-LIB efforts have the potential to improve the overall recovery by >25% (graphite, electrolyte and Li and transition metals).

### 2.2. ESCAPE Approach

The ESCAPE (Evaluation of Sustainability of material substitution using CArbon footPrint by a simplifiEd approach) approach was introduced by Bontempi [17]. The method is usually evaluated before the LCA: this was designed to allow saving time and resources if a certain technology at the lab scale proves to be inconvenient, thus making it pointless to extend the study to a more detailed analysis of the full life cycle [16].

The ESCAPE index allows for the evaluation of the sustainability of a process [17]. It was accounted by Equation (1):

ESCAPE index = [log (EE__raw_/(MJ/kg))−log(EE__sub_/(MJ/kg)) + log(CF__raw_) − log(CF__sub_)]/2 (1)

where: EE__raw_ is the embodied energy of the raw material in MJ/kg; EE__sub_ is the EE of the newly proposed material in MJ/kg. CF__raw_ is the CO_2_ footprint of the raw material in kgCO_2_/kg; CF__sub_ is the CF of the newly proposed material in kgCO_2_/kg.

The reference material (raw material) can be a primary raw material or a commercial product.

The ESCAPE approach was applied to ten processes reported in Table 1.

**Figure 1:**
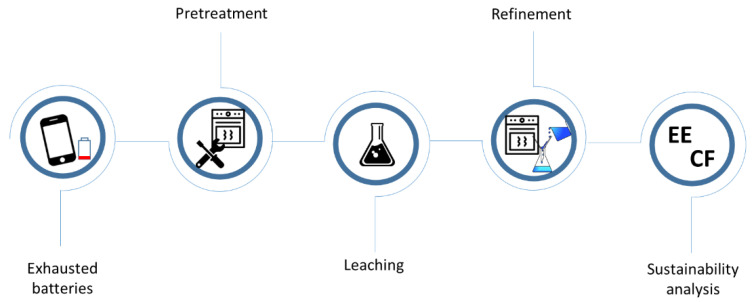
Schematic representation of the sustainability analysis in terms of Embodied energy (EE) and Carbon footprint (CF) of the metals extraction process.

As an example, a calculation performed for a technology that uses sulfuric acid as a leaching agent is reported and schematically represented in Figure 1 [21].

The process proposed by Ferreira et al. is a hydrometallurgical process in which the steps that make up the entire process were identified as follows:

1. The material used is made up of exhausted Li batteries from phones of different manufacturers and of different sizes. Manual dismantling of the battery, unrolling and separation of the layers for anode and cathode;
2. Drying of the layers for 24 h at 60 °C;
3. Shredded anode and cathode sheets using scissors;
4. Leaching in two steps: the first step involves the use of NaOH as a leaching agent and is repeated twice, to selectively leach Al; the second step involves the use of H_2_SO_4_ as a leaching agent and H_2_O_2_ as a reducing agent, and is always repeated twice, to leach Co and Li. The solutions are mechanically stirred with a helical impeller at a speed of 300 rpm. The optimal experimental conditions, which were taken as a reference for calculating the ESCAPE index, were determined with preliminary tests and are as follows:
  - Leaching with sodium hydroxide (t = 1 h, T = 50 °C, NaOH (*w*/*w*) = 10%, solid-liquid ratio (S/L ratio) = 1:10 g/mL for the first step, 1:30 g/mL for the second step);
  - Leaching with sulfuric acid (t = 1 h, T = 60 °C, H_2_SO_4_ (*w*/*w*) = 6%, S/L ratio = 1:30 g/mL, H_2_O_2_ (*w*/*w*) = 1%;
5. Crystallization in an oven at 60 °C to evaporate up to 95% of the volume of water;
6. Filtration of the remaining solution.

All calculations referred to 1 kg of BM material and in the final calculation, EE and CF referred to 1 kg of mineral (the choice is Co). For thermal treatment at low temperatures (around 100 °C), a power value of 400 W was used, while at high temperatures (>100 °C) 2500 W was used. The time not specified in the literature (or more than 24 h) was set to 1 h. This hypothesis is reasonable thinking to extend these processes on an industrial scale. For the drying process, the room temperature was considered, if not specified. For mechanical processes, the time was fixed at 5 min, if not specified. The water used for the preparation of solutions was distilled water, while tap water was considered for the washing steps. The quantity of water used was fixed at 10 L if not specified. For the battery discharge process if NaCl, NaOH, or Na_2_SO_4_ was at a low concentration if not specified it is assumed to use 10 L of solution. Instead, if no detail was given it was assumed that the batteries discharge spontaneously (EE and CF are 0).

In the case that H_2_O_2_ was used as a reducing agent unless specified in the article, it was assumed to use 30% of a commercial solution.

### 2.3. Model Validation

Validation is at the core of this methodology; this step shows the validity of the ESCAPE method applied to the LIBs recovery methods. This tool has already been adopted in other case studies [17,29] and to achieve this goal, the ESCAPE method was applied to calculate embodied energy (EE) and carbon footprint (CF) of the Co starting from the extraction of the metal from virgin rock proposed in the literature. The values obtained were then compared with those present in the Ecoinvent v.3.1 database. To carry out the validation, the process presented by Biswas and Bafubiandi [8] was chosen, which was an example of high-grade Co mining and refining, compared to other sources.

The starting mineral is an oxidized copper-cobalt mineral, from which Co is extracted by leaching with organic acids. The process is summarized in three steps:

1. Crushing of the ore with a jaw crusher;
2. Grinding of the ore with a roller mill;
3. Leaching of the mineral by citric acid.

The EE and CF values, referred to as 1 kg of Co and taken from the Ecoinvent v3.1 database, come from a study based on data provided by members of the Cobalt Development Institute and reflect current metal processing and extraction technologies. In this study, there are two main methods of Co extraction, namely hydrometallurgy and pyrometallurgy. The values of EE and CF associated with Co were obtained as a weighted average of different metal production processes, in which Co is obtained as a by-product of the extraction of Cu and Ni.

It has been hypothesized to carry out the calculation using, instead of citric acid, sulfuric acid, one of the most used acids during the extraction of Co from minerals by hydrometallurgy [30,31,32]. The EE and CF associated with sulfuric acid are significantly lower than those associated with citric acid, therefore the contribution given by the chemicals is significantly lower. The experimental conditions of leaching are as follows:

- H_2_SO_4_ concentration = 0.15 mol/L;
- Solid-liquid ratio = 20 g/L;
- Temperature = 80 °C;
- Duration of the process = 2 h.

Furthermore, since it is not specified in the article, the duration of crushing and grinding has been set equal to 5 min each.

Each contribution was then summarized, and the total was then multiplied by a conversion factor, which considers both the percentage of Co present in the mineral (as an average of the degree of purity given by the references in the aforementioned literature), and the recovery efficiency of the Co.

### 2.4. EE and CF Optimization

For the optimization of EE and CF, a process that used sulphuric acid and caustic soda as leaching agents [21] was considered. The choice was based on real experiments focused on the separation of Al, Co, and Li from spent Li-ion batteries and the main variables affecting the selective leaching of Al with NaOH. To consider that, leaching of Co and Li was studied with H_2_SO_4_. Regarding the ESCAPE approach, we defined three experimental conditions (NaOH at different L:S ratios and H_2_SO_4_ compared with NaOH), fixing the same boundary conditions for the calculation: time (t), temperature (T) and mixing speed (rpm). Detailed calculations are presented in Supplementary Material S1.

Some considerations were done based on the different energy supply mixes for each country; different ways to produce energy (fossil fuels or renewables) are available [33,34], and they must be considered to quantify accurately the EE and CF emissions to produce materials. I_EE_ and I_CF_ index (based on the EE and CF associated with the production of 1 kg of material) for thermal and mechanical processes were used as world average values, according to the different national energy mixes. These indexes change from country to country; therefore, a process environmental impact can depend on where it is carried out. Some considerations were done in the Results and Discussion paragraph, by fixing the entire configuration of ESCAPE calculation, and by only varying the energy mix (the location where the process is carried out physically). All the I_EE_ and I_CF_ indexes for the EU countries were reported in Supplementary Material S2.

### 2.5. Eco-Cost Evaluation

In this article, the eco-cost indicator is proposed to evaluate the virtual cost associated with emissions of a process [18]. In particular, the CF can be associated with global warming as indicated also in the LCA studies. Vogtländer reports that once the mass of CO_2_ potentially emitted per kg of material is known, CF (kgCO_2_/kgCo) can be multiplied by the conversion factor attributed to the global warming potential (0.114 EUR/kgCO_2_) with the results representing the eco-cost to get 1 kg of cobalt. This value, corresponding to an eco-cost, is considered to evaluate the sustainability of a process. To add that, there is a correction to consider which is the cumulative consumer price index (CPI) since 2000 (the date approximately in which Vogtländer elaborated data).

Therefore, for a more tangible EUR value, it is recommended to consider the inflation associated with the CPI from 2000 to 2021 (approximately 56% [35]). The eco-cost indicator system in the present study is in compliance with EN ISO 14008:2020 [36].

## 3. Results and Discussion

### 3.1. Model Validation

The major contributor (about 61%) derived from leaching with H_2_SO_4_ (EE and CF are 17.32 MJ/kg ore and 0.94 kgCO_2_/kg ore, respectively). This contribution was added to the leaching reactor (EE and CF are 9.19 MJ/kg ore and 0.54 kgCO_2_/kg ore, respectively), crushing (EE and CF are 0.75 MJ/kg ore and 0.04 kgCO_2_/kg ore, respectively), and milling (EE and CF are 1.23 MJ/kg ore and 0.07 kgCO_2_/kg ore, respectively) processes.

Considering the conversion factor, the final value of EE and CF is 608 ± 84 MJ/kg Co and 34 ± 4 kg CO_2_/kg Co, respectively.

**Figure 2:**
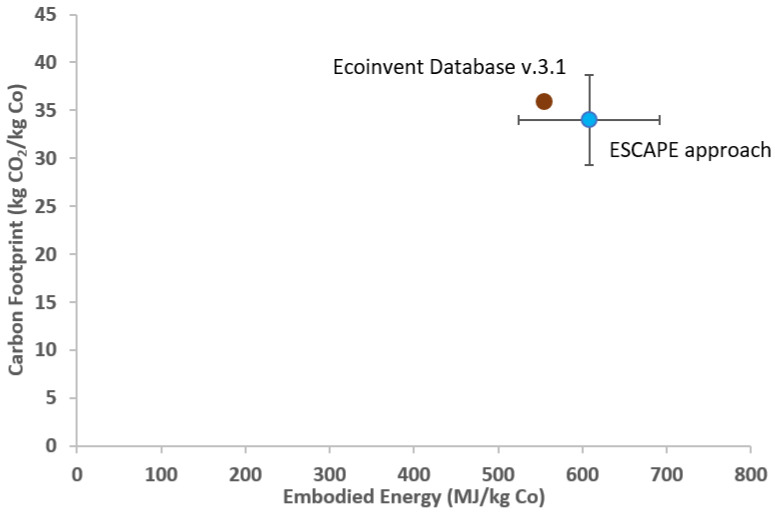
EE and CF of reference process evaluated by Ecoinvent Database and Escape approach for the process presented by Biswas and Bafubiandi [8].

Figure 2 shows the EE and CF of the reference process evaluated by ESCAPE approach and Ecoinvent v3.1. It can be noted that the results obtained by ESCAPE approach are similar to the values found with the Ecoinvent database (EE = 553.9 MJ/kg Co and CF = 35.98 kgCO_2_/kg Co).

The percentage variation (%) between the average EE value obtained by the ESCAPE approach and that provided by the database is 13.76%. This is an excellent result which supports the solidity of the calculation using the ESCAPE method, also considering that the value provided by the Ecoinvent database is a weighted average of various involved processes for the LCA study.

Furthermore, the obtained CF value differs from the one present in the database by 13.78%, which is indicative of how this parameter is related to EE. Assumptions had to be made to proceed with the calculation, such as the duration of the mechanical processes, and the fact that the related contribution to the transport was not considered.

### 3.2. Escape Approach

Considering the 4 contributions of EE and CF (mechanical, thermal, chemical and water), the total values of EE and CF associated with the recovery process (process n°2 of Table 1, using NaOH as a leaching agent) reported in Ferreira et al. [21] are:

EE__sub_ = 21.69 MJ/kg_BM

CF__sub_ = 0.58 kgCO_2_/kg_BM

The greatest contribution in terms of EE is given by the chemical contribution (EE = 12.22 MJ/kg_BM), while in terms of CF the prevailing contribution is the thermal one (CF = 0.52 kgCO_2_/kg_BM).

The result obtained was normalized to 1 kg of Co according to the following simple procedure:

- The percentage of Co with respect to the entire mass of the cathode is identified. In this case, the value is 43.3%; then we assume the BM contains 1:1 equally measured anode (graphite) and cathode so that means the % of Co in the BM is 21.65%.
- The conversion factor was identified by calculating the inverse of the % value of Co in the cathode. In this case, a conversion factor of 4.76 was obtained for the BM. Since the recovery percentage of Co is very high (97%), it was decided to ignore this detail in the calculation of the conversion factor, which would be very similar to the value just found;
- The conversion factor was then multiplied by the EE__SUB_ and CF__SUB_ results previously identified.

The result is:

EE = 103.3 MJ/kgCo__SUB_

CF = 2.77 kgCO_2_/kgCo__SUB_

The ESCAPE index of the process examined was equal to 0.92, therefore, a positive value indicates that the proposed alternative hydrometallurgical process is more sustainable than the reference process. This positive result is mainly attributed to the percentage of Co present inside the cathode which is much higher than that which can be found inside the virgin rock. 

The final EE and CF values evaluated for all the processes were reported in Table 2. The details of the EE, CF and ESCAPE index calculations are reported in Supplementary Material S1.

**Figure 3:**
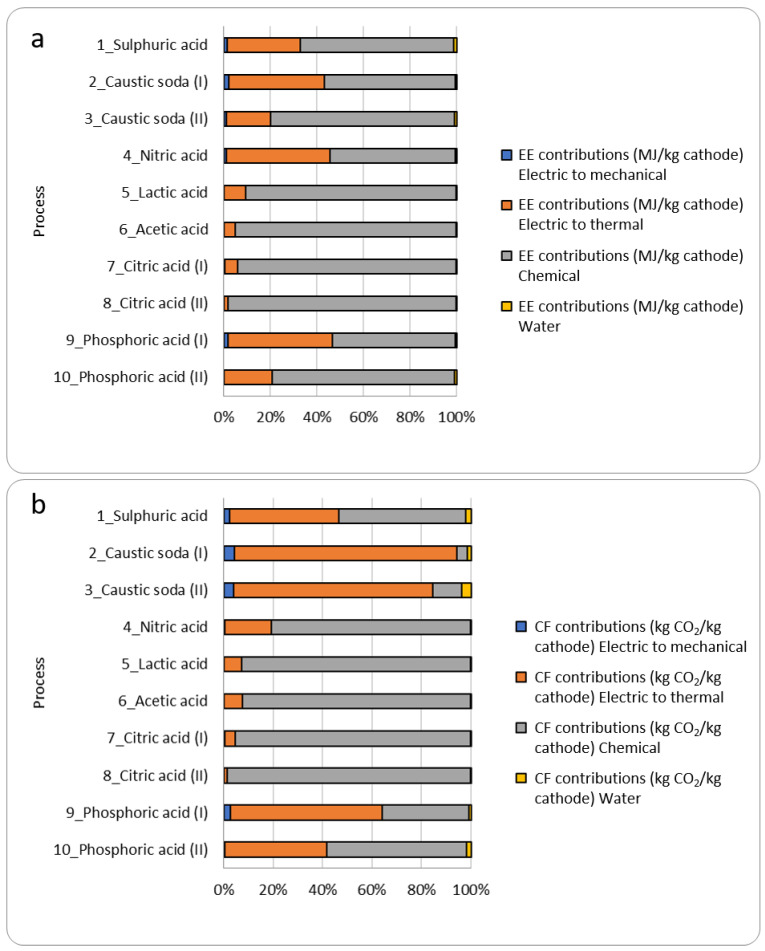
(**a**) EE; and (**b**) CF resulting from the 10 processes considered in this work, evaluating separately the contributions of technologies steps: mechanical, thermal, and chemical treatments and water usage.

Generally, EE is lower in processes that involve the use of inorganic rather than organic acids. This can be explained by comparing the EE values of the individual acids. Figure 3 shows EE (a) and CF (b) evaluated for 10 processes considering the 4 contributions (electric to mechanical, electric to thermal, chemical, and water).

EE of organic acids is greater than that of inorganic acids, and this, combined with the fact that the chemical contribution given by leaching is usually the prevailing one, ensures that the EE__SUB_ of processes that use organic acids is significantly higher. Given that EE citric acid = 85.66 MJ/kg, EE phosphoric acid = 27.17 MJ/kg, and CF citric acid = 6.14 kgCO_2_/kg, CF phosphoric acid = 0.38 kg CO_2_/kg. For instance, the study by Li et al. (process n°7) [25], shows that 92% of CF derives only from the chemical contribution. The mechanical contribution and that given by water are relatively low compared to the thermal and chemical ones (in particular, for the determination of C the contribution of water is zero). This is because although the operating powers of mechanical instruments are sometimes high, the time frame for electricity usage is generally lower for hydrometallurgical processes. The contribution of water is much lower than the other contributions given the assumption to use distilled water (EE = 0.01354 MJ/kg and CF = 0.0008 kgCO_2eq_/kg of water for the dilution and washing).

**Figure 4:**
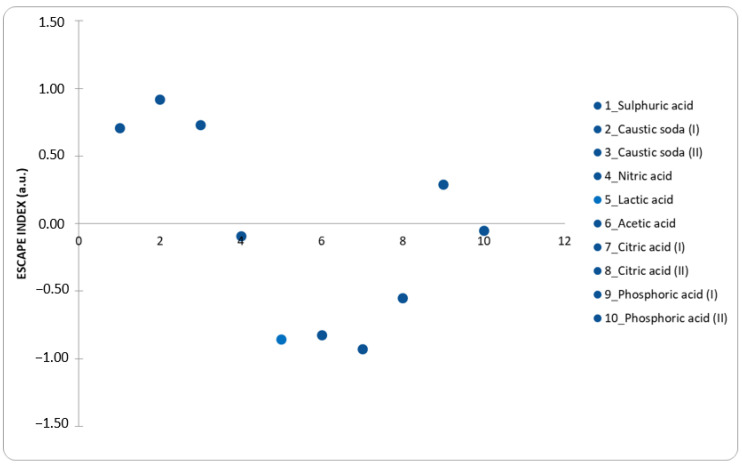
ESCAPE index values of 10 processes with related leaching agents, reported in Table 1.

As can be seen from Figure 4, the ESCAPE index values vary from a minimum of −0.93 to a maximum of 0.92, therefore a rather narrow range considering that this index extends up to values even close to 10, also considering that a step variation of 0.1 in the index corresponds to EE and CF variation of about 25% [29]. The ESCAPE calculations for all processes are reported in Supplementary Materials S1.

The processes (1, 2, 3, 9) all have a positive ESCAPE index value, which indicates that the recovery processes using inorganic acids in the leaching solution are more environmentally sustainable than the processes of extracting cobalt from virgin rock.

However, if the study will be extended to LCA analysis, organic acids should result more sustainable in comparison to inorganic ones, with a resulting relatively negligible impact.

Therefore, although certain processes are characterized by a negative ESCAPE index; at an industrial level it could be possible to approach “zero” by making improvements to the process, focusing on the part of the relevant contribution in terms of EE and CF. For example, in the study by Li et al. [25], the idea of recovering up to 90% of citric acid by lowering the pH of the solution itself and thus precipitating the metals contained in it was considered, then removed by filtration. Still, in the same study, it is suggested that the efficiency of the process could be further increased by lowering the calcination temperature, optimizing the concentrations of the reagents, and maximizing the recycling of acid. Even if there are no other references in this regard, it is not unlikely that the recycling of acid and the improvements mentioned above are also possible with other organic acids typologies; scientific research is moving in this direction.

### 3.3. Optimization

#### 3.3.1. Hydrometallurgical Setup

**Figure 5:**
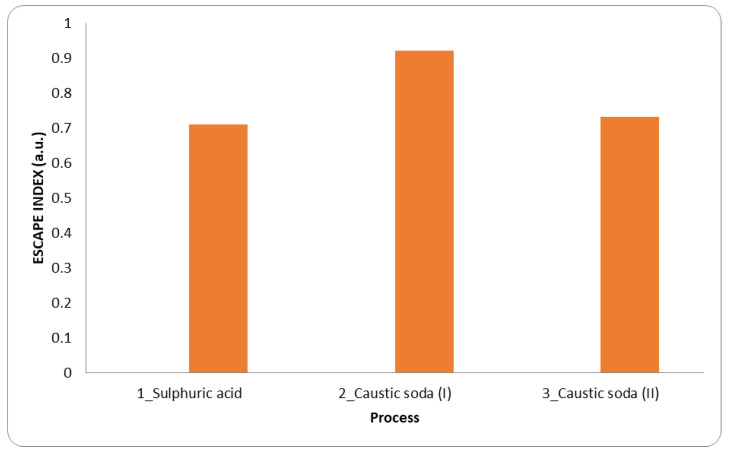
ESCAPE index for the three experimental conditions studied by Ferreira et al., 2008 [21]. It has been considered the case using H_2_SO_4_ at L/S = 30 (process n°1) and NaOH (2.5 M) with different L/S: equal to 100 (process n°2), and equal to 30 (process n°3).

Figure 5 represents the different ESCAPE indexes for each chemical condition reported in Ferreira et al., 2008 [21]. In all cases, the ESCAPE index remains positive and varies between 0.71 and 0.92. The most environmentally friendly condition is set in the case of using NaOH (2.5 M) at S/L ratio equal to 100 g BM to L leaching solution. The major presence of input material (the end-of-life BM) makes the process more advantageous for the calculation. The main contribution at this condition is the chemical used (12.22 MJ/kg BM and 0.03 kgCO_2_/kg BM) and the same goes for process 3 (S/L equal to 30), but differing threefold in terms of chemical contribution (36.67 MJ/kg BM and 0.08 kgCO_2_/kg BM). The electricity contribution does not change (8.9 MJ thermal and 0.44 MJ mechanical energy, 0.52 kgCO_2_ thermal and 0.03 kgCO_2_ mechanical emission) due to the boundary system remaining fixed under the initial set of calculation as reported in the methods section. The change of leaching agent (from alkaline to acidic) does not vary the outcome of the impact (considering the same S/L ratio, the ESCAPE index is equal to 0.73 compared to 0.71 for NaOH and H_2_SO_4_, respectively). In terms of contribution, the water is comparable (0.387 MJ of process 3 against 0.393 MJ). On the contrary, due to the less use of H_2_SO_4_ than NaOH for the different concentrations (22 mol/kg BM against 75 mol/kg BM, respectively), the EE chemical contribution is 18.67 MJ vs. 36.67 MJ). Instead, the CF chemical contribution is more impactful for the acidic reagent (0.61 kgCO_2_ vs. 0.08 kgCO_2_ of NaOH case) despite it being used more NaOH in terms of input mass.

#### 3.3.2. Geographical Dependence

**Figure 6:**
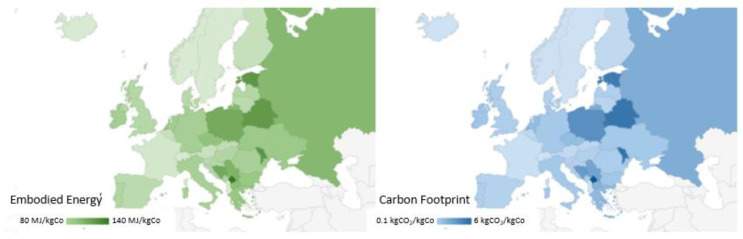
Evaluation of Embodied Energy and Carbon Footprint for Co extraction process by Ferreira et al., 2008 (process n°2 of Table 1) [21], according to the national energy mix.

Figure 6 shows the results obtained by applying the ESCAPE method, changing the I_EE_ and I_CF_ indices for mechanical and thermal processes depending on the country where the process may be applied, for process n°2 of Table 1 (NaOH with L/S equal to 100). Figure 6 shows that there is a huge differentiation of impacts region by region. We move from more negatively impactful Eastern countries where there is more dependence on carbon-based fossil fuel in their energy mix to a lower impact where nuclear and renewables (eolic, hydrothermal, geothermal) have a major influence. This difference is clear just by comparing the cases for hydrometallurgical processes which are characterized by more use of chemicals and water in the flow sheet of their process. Therefore, if we consider a process involving more thermal energy with consequent higher T, the choice of where to apply a certain process is crucial.

**Figure 7:**
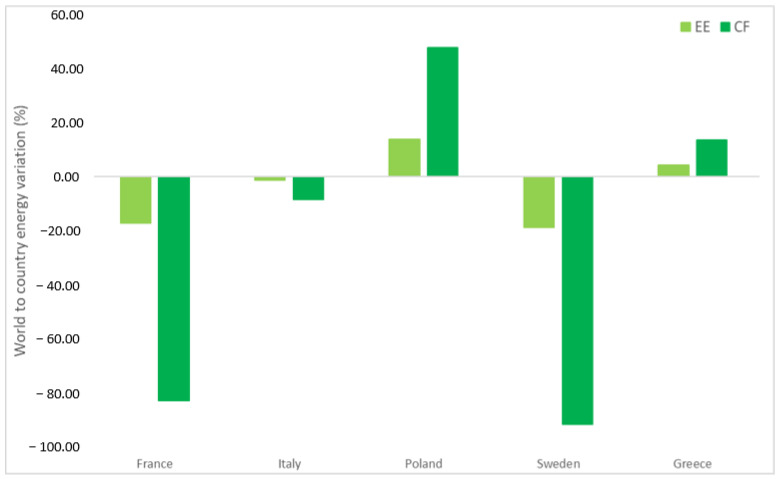
Variation (%) of EE and CF compared to world averaged values. Data refer to the process based on the use of sulfuric acid [21].

As expected, for instance, the values of EE and CF change according to the country considered and more favorable values are obtained in the case of countries with energy mixes mainly consisting of low-impact sources (such as Sweden and France). This is synonymous with a sustainable process, and the same consideration is taken for Italy in less measure. This difference can be better appreciated in Figure 7, where we have chosen to focus again on the sulfuric acid-based process [21]. They have a lower impact relative to the calculation based on the world average index, calculated as a mean value considering all the countries across the globe. This relative difference is highlighted in the carbon footprint emission (less than 90% of the world average for France and Sweden). The world index bears the presence of very dense populations (United States of America, India, and China) that are responsible for a huge part (more than half of emitting CO_2_ from fossil fuel) of the global warming occurring currently [37].

On the contrary, for example, among the most “emitters” there are Poland and Greece, with I_EE_ and I_CF_ values higher than the global average by 50% in the case of Poland. More mitigated effects are found regarding the EE that sets between plus and minus 15% for the countries discussed above. The EE and CF values for all EU countries are reported in Supplementary Material S2.

### 3.4. Eco-Cost Evaluation

**Figure 8:**
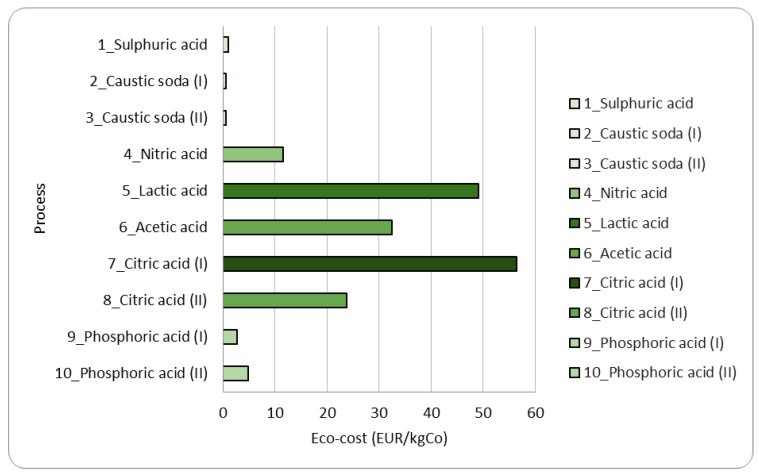
Eco-cost results calculated for the 10 processes reported in Table 1.

Figure 8 reports the eco-cost results for the 10 processes reported in Table 1.

In line with what is obtained by quantifying the CF, here we have a high prevention cost for the processes using organic acids as leaching agents (acetic acid, lactic acid, and citric acid) as indicated in Figure 8. This means there is a need for more incentives to scale up the technology. This stands regardless of the negligible emissions occurring using organic acids. In the case of inorganic acids, this marginal cost is much reduced. It is worth appraising that this kind of information could discourage a process designer since we are talking about flow sheets meant to work at a laboratory scale. To put better into investment perspective at a world scale, to prevent 1000 kgCO_2_ emission, one, for instance, should invest €116, in offshore windmill farms (and the other CO_2_ reduction systems at that price or less) [38]. When this is done consequently, and all possible prevention measures which are less expensive are taken as well, the total CO_2_ emissions in the world would be reduced by 70% compared to the emissions in 2008. As a result, global warming will stabilize [39], and the information coming out of this study must be interpreted in a global sense.

Another reason to take into account the importance of this indicator is related to carbon pricing; this is an instrument that captures the external costs (as mentioned above like the case of eco-cost) of GHG emissions—the costs of emissions that the people pay for, such as damage to crops, health care costs from heat waves and droughts, and loss of property from flooding and sea level rise—and ties them to their sources through a price, usually in the form of a price on the CO_2_ emitted. A price set on carbon pushes for a shift of the burden for the damage from GHG emissions back to those who are responsible for it and identifying who can avoid it. Instead of dictating who should reduce emissions, a carbon price can provide an economic signal to emitters and allows them to decide to either transform their activities (in this case developing alternative processes for extracting a critical raw material) and lower their emissions or continue emitting and paying for their emissions. Placing a proper price on GHG emissions is of fundamental importance to internalize the external cost of climate change in the broadest possible range of economic decision-making and in fixing economic incentives in terms of investments in favor of the clean technologies transition [40].

There is a growing consensus among both businesses and governments on the role of carbon pricing in the transition to a decarbonized economy. For governments, carbon pricing is one of the instruments of the climate policy package needed to reduce emissions and as a possible source of revenue.

## 4. Conclusions

In the present work, different hydrometallurgical processes for the recovery of metals from discarded lithium batteries were compared to evaluate their sustainability. For this aim, the ESCAPE approach, a new and simplified tool, was used and its potentialities were demonstrated. The results show that the choice of leaching processes based on inorganic acids is currently the most suitable possibility for the recovery of metals from LIBs. It is desirable, however, in the future to invest more in developing technologies based on the use of organic acids, because the ESCAPE index, used to compare different processes, is always very close to zero, indicating that a slight process improvement may increase its sustainability. This may be achieved, for example, through the recovery and reuse of organic leaching solutions. In conclusion, it was shown that considering their low material and management costs, the recycling processes could be less impactful in comparison to extracting metals from the corresponding ores. In addition, the location to carry out the LIBs recovery processes is not indifferent, because the embodied energy and carbon footprint values vary from country to country depending on the national energy mix. Finally, the evaluation based on eco-costs reveals that the use of organic acids in hydrometallurgical methods to recycle critical metals from spent LIBs could be more costly (approximately fivefold) than the average use of inorganic acid.

## Figures and Tables

**Table 1:** Processes studied for Co extraction by means of different leaching agents.

Process Number	Leaching Agent	Reference
1	Sulphuric acid	[21]
2	Caustic soda (I)	[21]
3	Caustic soda (II)	[21]
4	Nitric acid	[22]
5	Lactic acid	[23]
6	Acetic acid	[24]
7	Citric acid (I)	[25]
8	Citric acid (II)	[26]
9	Phosphoric acid (I)	[27]
10	Phosphoric acid (II)	[28]

**Table 2:** EE and CF evaluated for all Co extraction processes considered in this paper.

Process Number	Leaching Agent	EE (MJ/kgCo)	CF (kgCO_2_/kgCo)
1	Sulphuric acid	135	5.6
2	Caustic soda (I)	103	2.8
3	Caustic soda (II)	221	3.1
4	Nitric acid	468	65
5	Lactic acid	3703	276
6	Acetic acid	4859	183
7	Citric acid (I)	4477	317
8	Citric acid (II)	1888	134
9	Phosphoric acid (I)	348	15
10	Phosphoric acid (II)	935	27

## Data Availability

Data are reported in Supplementary Materials S1 and S2.

## Supplementary Materials

The following supporting information can be downloaded at: https://www.mdpi.com/article/10.3390/ma15238527/s1, Supplementary Material S1: ESCAPE approach evaluated for ten Co extraction processes and Supplementary Material S2: EE and CF evaluation based on geographical dependence”. Refs [21,22,23,24,25,26,27,28] are cited in Supplementary Materials.
